# TDDFT Study on the ESIPT Properties of 2-(2′-Hydroxyphenyl)-Benzothiazole and Sensing Mechanism of a Derived Fluorescent Probe for Fluoride Ion

**DOI:** 10.3390/molecules29071541

**Published:** 2024-03-29

**Authors:** Tingting Wang, Meiheng Lv, Yuhang Zhang, Yue Gao, Zexu Cai, Yifan Zhang, Jiaqi Song, Jianyong Liu, Hang Yin, Fangjian Shang

**Affiliations:** 1 College of Science, Shenyang University of Chemical Technology, Shenyang 110142, China; wangtingting_7@163.com (T.W.); zyh10115@163.com (Y.Z.); 18640119996@163.com (Y.G.); 13390210268@163.com (Z.C.); 15114233893@163.com (Y.Z.); 13130927655@163.com (J.S.); 2 Research Center of Advanced Biological Manufacture, Dalian National Laboratory for Clean Energy, Dalian Institute of Chemical Physics, Chinese Academy of Sciences, Dalian 116023, China; yinhang@cjlu.edu.cn; 3 College of Aeronautical Engineering, Binzhou University, Binzhou 256603, China; shangfangjian@bzu.edu.cn

**Keywords:** hydrogen bond, ESIPT, fluorescent probe, sensing mechanism, F^−^ detection

## Abstract

The level of fluoride ions (F^−^) in the human body is closely related to various pathological and physiological states, and the rapid detection of F^−^ is important for studying physiological processes and the early diagnosis of diseases. In this study, the detailed sensing mechanism of a novel high-efficiency probe (PBT) based on 2-(2′-hydroxyphenyl)-benzothiazole derivatives towards F^−^ has been fully investigated based on density functional theory (DFT) and time-dependent density functional theory (TDDFT) methods. F^−^ attacks the O-P bond of PBT to cleavage the dimethylphosphinothionyl group, and the potential products were evaluated by Gibbs free energy and spectroscopic analyses, which ultimately identified the product as HBT-Enol1 with an intramolecular hydrogen bond. Bond parameters, infrared vibrational spectroscopy and charge analysis indicate that the hydrogen bond is enhanced at the excited state (S_1_), favoring excited state intramolecular proton transfer (ESIPT). The mild energy barrier further evidences the occurrence of ESIPT. Combined with frontier molecular orbital (FMO) analysis, the fluorescence quenching of PBT was attributed to the photoinduced electron transfer (PET) mechanism and the fluorescence turn-on mechanism of the product was attributed to the ESIPT process of HBT-Enol1.

## 1. Introduction

The detection of anions has attracted a great deal of attention due to the important role they play in chemical, environmental and biological systems [1,2,3]. Fluoride ion (F^−^) is the smallest and most electronegative anion, and it holds significance in various fields such as food science, dental care and osteoporosis treatment [4,5,6,7,8,9]. Moderate intake of F^−^ is beneficial to health, but improper intake (excessive or chronic deficiency) can cause a variety of diseases. For example, large doses of F^−^ may cause acute stomach and kidney problems in humans [10,11]. The World Health Organization (WHO) has established a maximum limit of 1.5 mg L^−1^ (79 μmol L^−1^) for F^−^ in drinking water [12]. Therefore, the development of efficient and reliable methods for the detection of F^−^ in chemical and biological systems has received increasing attention.

So far, many conventional methods have been used to detect F^−^, such as ion-selective electrode [13], ion chromatography [14] and the colorimetric method [15], but these methods have the disadvantages of having complicated handling of samples, expensive instruments and long detection time. On the contrary, the fluorescent probe method [16,17,18,19] has the advantages of short response time, real-time monitoring, low cost and strong naked-eye detection ability. Depending on the intermolecular forces, the sensing mechanisms of F^−^ recognition can be classified into four categories: (1) hydrogen bond interactions [20,21,22]; (2) Lewis acid–base interactions [23]; (3) anion–π interactions [24,25]; (4) F^−^ induced chemical reactions [26,27,28].

In recent years, many researchers have conducted extensive and detailed studies on the different types of fluorescent probes for F^−^. Gu et al. [29] in 2019 reported a fluorescent probe with tert-butyldimethylchlorosilane as the detection group, which can be used for the dual detection of fluoride ions and mercury ions. The probe molecule had no fluorescence emission under aqueous environment, whereas the addition of fluoride anion would result in the generation of hydroxythiocoumarin with strong fluorescence emission. Chen et al. [30] reported a fluorescent probe for F^−^ containing a 1,8-naphthimide structure, which utilizes the N-H bond in the stilbene structure to form a hydrogen bond with F^−^. In addition to these experimental studies on F^−^ fluorescent probes, a number of scholars have conducted theoretical computational studies on F^−^ detection. Yang et al. [31] investigated the sensing performance of a newly synthesized fluoride sensor, 2-[2-(tert-butyl-diphenyl-silanyloxy)-phenyl]-4,5-diphenyl-1H-imidazole, based on the first principle calculations, and the sensing performance is extremely mechanistic. The fluoride ions in the ground state triggered a cleavage reaction of the Si-O bond, changing the absorption spectrum of the probe. And the fluorescent change attributes to a PET process. Kediya et al. [32] studied the F anion sensing mechanism of a fluorescent probe *N*-(2-(benzothiazole-2-yl)phenyl)-4-methoxybenzamide (probe4). In the ground state, the F anion interacts via the F^−^⋯H-N bond to deprotonate the amide proton, resulting in a distorted geometry. After deprotonation, significant intramolecular charge transfer (ICT) features were observed in probe4, thereby observing a redshift in the absorption and emission spectra.

Numerous studies in recent years have shown that ESIPT occurs when an excited state is transferred from a proton donor to an adjacent proton acceptor via hydrogen bonding [33,34,35,36,37,38,39,40]. The ESIPT process usually produces large Stokes shifts and enhances the photochemical stability of the molecule, making it suitable for fluorescent probes [41,42,43]. Zhang et al. [44] investigated a reversible proportional fluorescent probe BT-Se based on intramolecular proton transfer in the excited state for the detection of HClO and H_2_S. When BT-Se detects HClO, it will be oxidized to BT-SeO; BT-SeO can be reduced to BT-Se by detecting H_2_S. The attribution of fluorescence peaks and the luminescence mechanism were studied in detail in the article. The results show that the excited state is more susceptible to the ESIPT process, with yellow and blue fluorescence emitted from the ketone structure after the proton transfer of BT-Se and BT-SeO, respectively. In addition, Tang et al. [45] investigated the sensing mechanism of a newly synthesized imidazo [1,5-α] pyridine-based fluorescent probe (MZC-AC) based on intramolecular proton transfer in the excited state for the detection of Cys. The fluorescence quenching mechanism of MZC- AC and the ESIPT process of MZC are explained in detail. The calculated potential energy curves demonstrate the difficulty of the ESIPT process occurring in MZC, and the results show that the fluorescence enhancement mechanism of MZC is not based on ESIPT. 2-(2′-hydroxyphenyl)-benzothiazole as a common ESIPT fluorophore, noted for its unique nature. Its optical properties have been clearly elaborated, and it exhibits a large Stokes shift due to the process of internal proton transfer in its enolitic excited state molecules. Many scholars have conducted a series of studies utilizing this feature. Zhang et al. [46] developed an ESIPT-based mitochondria-targeted proportional fluorescent bioprobe (HBTP-mito) based on a phosphorylated 2-(2′-hydroxyphenyl)-benzothiazole derivative for ALP detection and visualization. The probe has good water solubility and fluoresces green in aqueous buffer due to ESIPT plugging. In the presence of ALP, the phosphate is hydrolyzed, the ESIPT process is resumed, and the fluorescence changes from green to red. The excited state intramolecular proton transfer mechanism of 2-(2′-hydroxyphenyl)-benzothiazole (HBT) derivatives obtained by altering the neighboring functional group of the hydroxyl group was investigated by Ji et al. [47]. The results show that hydrogen bonding is enhanced in the first excited state, and HBT and its derivatives are more susceptible to ultrafast ESIPT behavior in the excited state. The excited state HBT derivatives have electrons with groups in the neighboring positions, weakening the intramolecular hydrogen bonding O-H⋯N to the detriment of the ESIPT process.

The excited state processes are closely related to the structure and fluorescence properties of the probe molecules, and more and more fluorescent probes based on ESIPT behavior are being designed for F^−^ detection [48]. For example, Du et al. [49] developed a fluorescent probe, PBT, based on 2-(2′-hydroxyphenyl)-benzothiazole (HBT) derivatives for the detection of F^−^. The dimethylphosphinothionyl group in PBT can effectively cause fluorescence quenching of the probe by disrupting the intramolecular hydrogen bond. However, Du et al. did not give a detailed explanation of the reaction mechanism of the probe PBT with F^−^, and the fluorescence mechanism of the system has not yet been clarified, which hinders the research on sensing and detection mechanisms, as well as the improvement and design of efficient fluorescent probes in the future. Thus, in this work, we used a high-precision theoretical calculation method to study the system in depth [50]. We have theoretically investigated the recognition and fluorescence mechanism of probe PBT for the detection of F^−^. Several relevant products after the reaction of probe PBT with F^−^ were investigated by DFT and TDDFT methods, and the rationality of the products was evaluated based on electronic spectra. Based on the optimized ground state (S_0_) and excited state (S_1_) configurations, bond length and bond angle data were obtained to study the intramolecular hydrogen bond changes. In addition, we performed infrared vibrational frequency analysis (IR), natural population analysis (NPA) and Gibbs free energy analysis before and after the excited state proton transfer to provide an in-depth investigation of the proton transfer phenomenon present in the products. Finally, we determined the fluorescence mechanism of the system by frontier molecular orbital analysis.

## 2. Results and Discussion

### 2.1. Geometric Configuration 

**Figure 1:**
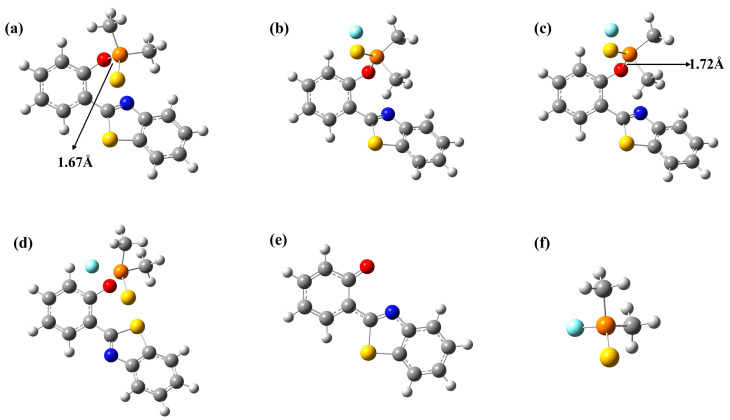
Ground state optimized configurations. (**a**) PBT, (**b**) TS1, (**c**) IM, (**d**) TS2, (**e**) HA^−^, (**f**) TP. Atoms with colors are C (gray), H (white), O (red) and N (blue). Important bond length data have been labeled in the figure.

The optimized ground state geometric configurations are shown in Figure 1, and the corresponding chemical structures are presented in Figure S1. Figure 1a shows the optimized configuration of the fluorescent probe PBT in the ground state. Figure 1b–d show the first transition state structure TS1, the intermediate structure IM and the second transition state structure TS2. Figure 1e,f show HA^−^ and TP, the products of PBT’s reaction with F^−^.

### 2.2. Energy Analysis

**Figure 2:**
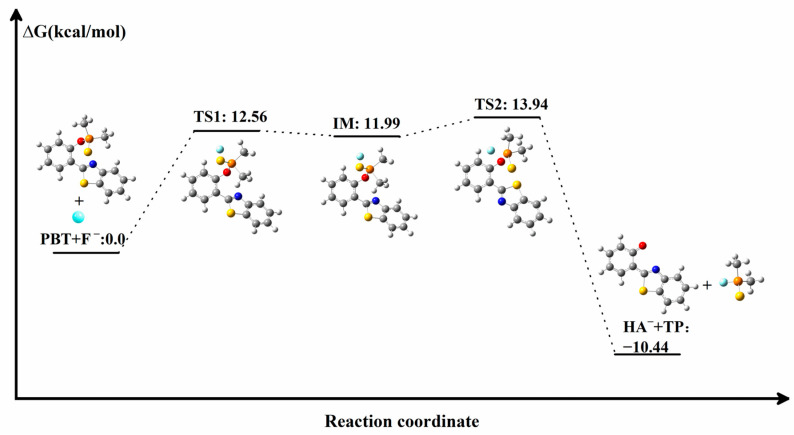
Gibbs free energy change (kcal/mol) for the PBT+F^−^ recognition process. IM: intermediate, TS: transition state. Atoms with colors are C (gray), H (white), O (red), N (blue), P (orange), S (yellow) and F (cyan).

In order to further elucidate the binding process of PBT to F^−^, we calculated the change in the Gibbs free energy of the system during the formation of the products HA^−^ and TP from the binding of PBT to F^−^. All energy data given in Figure 2 are relative energies calculated based on the sum of the energies of the reactants PBT and F^−^. As shown in Figure 2, the reaction between PBT and F^−^ is a two-step reaction; first, F^−^ is close to PBT through the transition state TS1 to form the intermediate IM. In the structure of IM, F^−^ is attached to P atom to form a P-F covalent bond; although there is no bond between the F^−^ and P atoms in the figure, we have analyzed the bond order between them, and it is found that their FBO bond order is 0.843, which is consistent with the range of single bond lengths. In addition, the distance between these two atoms is 1.96 Å, which also falls within the range of bond between P and F^−^, so a relatively weak bond is formed between P and F^−^. As shown in Figure 1a, in PBT, the P-O bond length is 1.67 Å. When F^−^ is attached to the P atom, the P-O bond length in the IM structure becomes 1.72 Å; the increase in the bond length indicates that the addition of F^−^ weakened the P-O bond. Consequently, with IM as the starting point, the P-O bond is subsequently broken through TS2, while two independent products, HA^−^ and TP, are finally formed. Since the F^−^ added in the original reaction is negatively charged, TP is neutral in the final product and HA^−^ is present as a negatively charged anion. The energy barrier from the intermediate IM to the transition state TS1 is 0.57 kcal/mol, and the energy barrier from the intermediate IM to the transition state TS2 is 1.95 kcal/mol, and both energy barriers are easy to cross. Although the reaction of PBT and F^−^ is a two-step reaction, the energies of IM and TS1 TS2 are close; IM is a relatively easy intermediate to cross, so the two-step reaction is a successive and relatively fast reaction. The reverse energy barrier from the products to the transition state TS2, however, is 24.38 kcal/mol, which is difficult to cross at room temperature. For the whole reaction, the sum of the relative energies of the products HA^−^ and TP is 10.44 kcal/mol, lower than that of the reactants, which ensures that the process proceeds thermodynamically favorably. The mild reaction energy barrier (13.94 kcal/mol) indicates that the process is kinetically permissible and the reaction is capable of proceeding at room temperature. Therefore, the reaction of PBT+F^−^ is a feasible process to recognize.

### 2.3. Electron Spectrum

**Figure 3:**
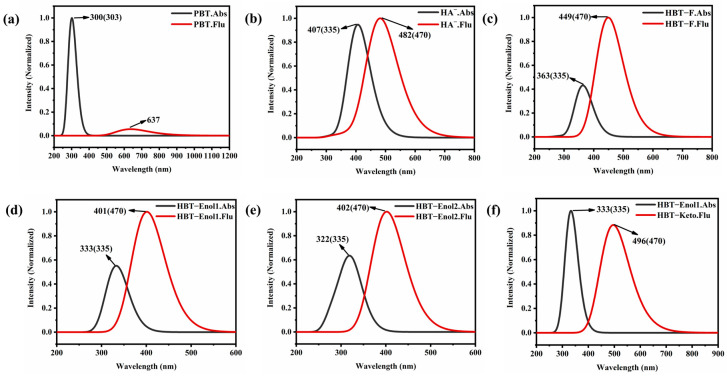
Electronic spectra of (**a**) PBT; (**b**) HA^−^; (**c**) HBT-F; (**d**) HBT-Enol1; (**e**) HBT-Enol2; (**f**) HBT-Keto. Important absorption and emission wavelength (nm) data have been labeled in the figures, and the corresponding experimental data are given in parentheses.

In order to check the reasonableness of the calculations and products, we obtained the UV absorption and emission spectra of the PBT and the product HA^−^ by fitting a Gaussian model, as shown in Figure 3a,b. The main absorption peak of the probe PBT molecule is located at 300 nm, which well reproduces the experimental results (303 nm). In addition, the oscillator strength of the maximum emission peak of the PBT molecule is almost zero, which is also consistent with the fluorescence quenching observed in the experiment. The high agreement between the calculated and experimental spectra confirms the rationality and reliability of our computational method. Comparing the absorption and emission values of the fluorescent product HA^−^ with the experimental values after the addition of F^−^, it can be found that the absorption value of HA^−^ is much larger than that in the experiment, with a redshift of 72 nm, so HA^−^ may not be the final reaction product.

**Figure 4:**
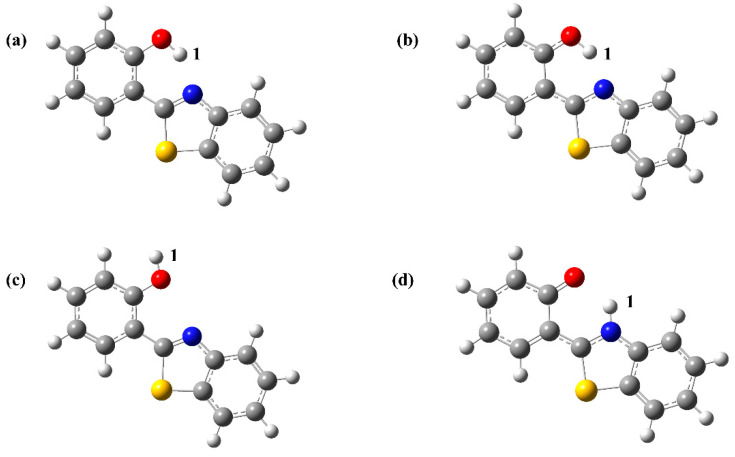
Optimized geometrical configurations of HBT-Enol1 in the ground and excited states, HBT-Enol2 in the ground state and HBT-Keto in the excited state. (**a**) HBT-Enol1(S_0_), (**b**) HBT-Enol1(S_1_), (**c**) HBT-Enol2(S_0_), (**d**) HBT-Keto(S_1_). Atoms with colors are C (gray), H (white), O (red), N (blue) and S (yellow).

Previous studies have shown that a new intermolecular hydrogen bond O⋯H-F will be formed based on the intramolecular hydrogen bond O-H⋯N in 2-(2′-hydroxyphenyl)-benzothiazole derivatives due to the addition of F^−^ [51]. Thus, we also designed the reaction pathway for the formation of a hydrogen bond between the anionic HA^−^ hydrated structure, HBT-Enol1 (Figure 4a) and F^−^. The optimized ground state structure of the product HBT-F is shown in Figure S2. To verify the plausibility of this reaction channel, we calculated the Gibbs free energy change for the HBT-Enol1+F^−^ recognition process, as shown in Figure S3, where the transition state is presented during O-H bond cleavage and F-H bond formation. The results show that the lower reaction energy barrier (3.81 kcal/ mol) allowed the reaction to occur rapidly. Meanwhile, the product energy is lower than the reactant energy, indicating that HBT-F is a stable product. In order to determine whether HBT-F is a reasonable product, we conducted a spectral analysis of it, as shown in Figure 3c; similar to HA^−^, the absorption value of HBT-F is redshifted compared to the experiment, so HBT-F is still not a desirable product.

Subsequently, we considered two other products of the hydrated structure, HBT-Enol1 and HBT-Enol2, and the optimized ground and excited state configurations were obtained as shown in Figure 4, with Figure 4a,b showing the stabilized configurations of HBT-Enol1 in the ground and excited states, respectively, and Figure 4c showing the stabilized configuration of HBT-Enol2 in the ground state. The UV absorption and emission spectra of HBT-Enol1 and HBT-Enol2 are shown in Figure 3d,e, respectively, from which it can be seen that the absorption–emission values of HBT-Enol1 and HBT-Enol2 are basically similar, and the absorption values are in high agreement with the data in the experiments as compared with the original experiments, whereas the fluorescence–emission values are smaller than the experimentally measured values, therefore HBT-Enol1 and HBT-Enol2 may not be the reasonable emission products with respect to the emission spectra. It is noteworthy that HBT has a potential proton transfer site due to the formation of an intramolecular hydrogen bond between N and O-H_1_ in the HBT-Enol1 conformation of this system, so the emission spectra may be due to the contribution of the HBT-Keto conformation (Figure 4d) after proton transfer. So, the following part mainly focuses on the proton transfer process of HBT-Enol1.

### 2.4. Excited State Proton Transfer

Important bond length and bond angle data related to the hydrogen bond of HBT-Enol1 and HBT-Keto are listed in Table 1. For HBT-Enol1, the bond length of the hydrogen bond H_1_⋯N is shortened from 1.718 Å in the S_0_ state to 1.638 Å in the S_1_ state, suggesting that the intramolecular hydrogen bond is strengthened in the S_1_ state. In addition, the increase in the hydrogen bond angle O-H_1_⋯N is further evidence of the hydrogen bond strengthening phenomenon in the S_1_ state.

**Figure 5:**
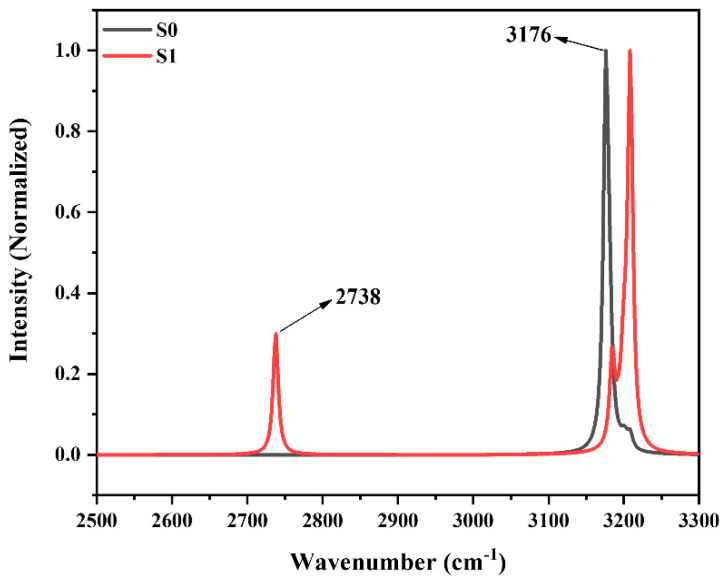
Calculated infrared vibrational spectra of O-H bonds in S_0_ and S_1_ states.

Infrared vibrational frequency analysis is an effective method to study the variation of the hydrogen bond [52]; Figure 5 gives the absorption peaks of the IR spectra of covalently bonded O-H_1_ of HBT-Enol1 in S_0_ and S_1_ states. It can be seen that the frequency of the O-H_1_ stretching vibration undergoes a significant redshift from 3176 cm^−1^ in S_0_ to 2738 cm^−1^ in the S_1_ state, suggesting a strengthening of the hydrogen bond of HBT-Enol1 in the excited state.

To further determine the accuracy of the hydrogen bond change results, we performed NPA charge analysis of HBT-Enol1 in the S_0_ and S_1_ states, as shown in Table S1. In the S_0_ state, the NPA charges of the O and N atoms of HBT-Enol1 are −0.662 and −0.486. When excited to the S_1_ state, the NPA charges of the O and N atoms are −0.656 and −0.496. It can be seen that photoexcitation increases the negative charge of the N atom of HBT-Enol1 and enhances the attraction to hydrogen, which promotes the proton transfer process. Hence, the proton transfer process of this system is more likely to occur in the S_1_ state.

**Figure 6:**
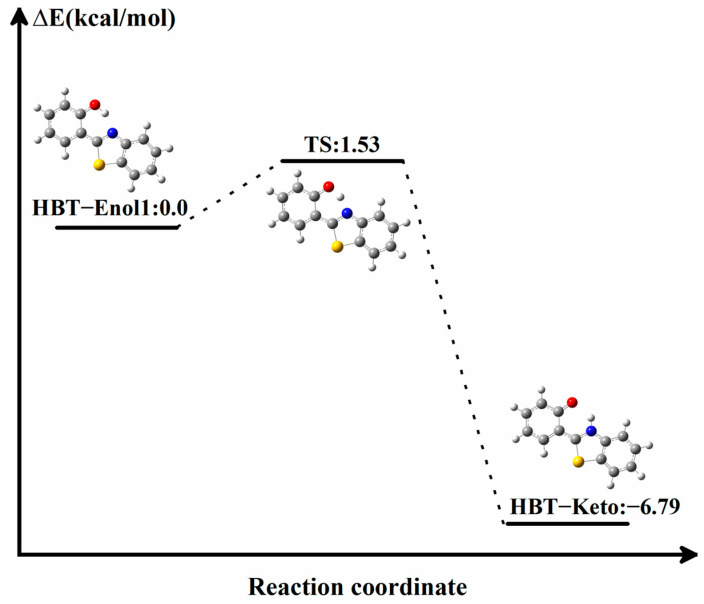
Electronic energy data for the conformational transitions of HBT-Enol1 and HBT-Keto. TS: transition state. Atoms with colors are C (gray), H (white), O (red), N (blue) and S (yellow).

Furthermore, in order to determine whether HBT-Enol1 will have the ESIPT process in the S_1_ state, the electronic energy of the proton transfer process was calculated by identifying the corresponding transition state. According to the results shown in Figure 6, first, the reaction is exothermic and the reaction drive is sufficient. Secondly, the mild reaction energy barrier makes it easy for the reaction to take place. In summary, we believe that HBT-Enol1 does exhibit ESIPT behavior in the excited state in this system, which is also consistent with the experimental results derived by previous authors. So, the fluorescence could be caused by the HBT-Keto configuration after proton transfer. The emission spectrum of the HBT-Keto is shown in Figure 3f; it can be seen that the HBT-Keto fluorescence emission spectral data are in good agreement with the experimental values. To summarize, the absorption data of the product in the experiments (335 nm) are mainly contributed by the HBT-Enol1 conformation, whereas the fluorescence emission value (470 nm) is contributed by the HBT-Keto conformation in the excited state after the proton transfer.

### 2.5. Sensing Mechanism

**Figure 7:**
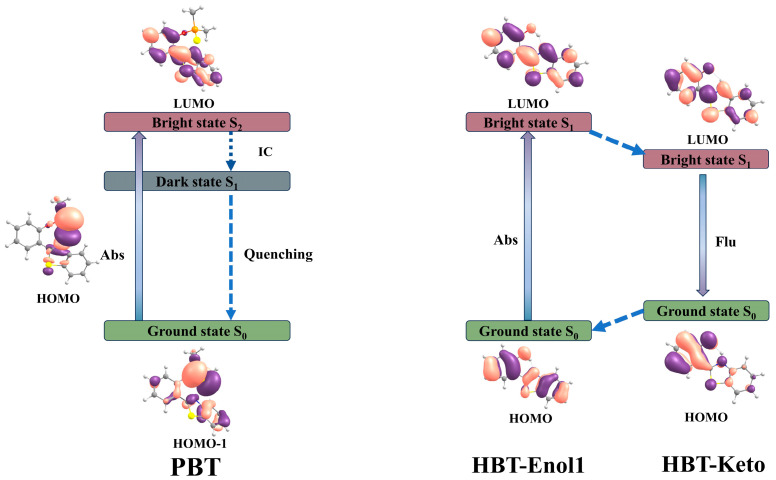
Schematic representation of the sensing mechanism and associated frontier molecular orbitals of PBT (**left**), HBT-Enol1(**right**) and HBT-Keto (**right**). Atoms with colors are C (gray), H (white), O (red), N (blue), P (orange) and S (yellow).

In order to elucidate the mechanism of sensing and detection of F^−^ by the fluorescent probe PBT, the electron transition and frontier molecular orbitals of PBT, HBT-Enol1 and HBT-Keto have been analyzed. The frontier molecular orbital (FMO) analysis diagrams are shown in Figure 7, and the corresponding electron transition data are given in Table 2. As shown on the left side of Figure 7, the absorption peaks of PBT can be attributed to the S_0_-S_2_ transition, which is mainly contributed by the electron transition between HOMO-1 and LUMO (Table 2). After the PBT molecule is excited to the S_2_ state, it will reach the S_1_ state through an ultra-fast internal conversion process, and the S_1_-S_0_ transition is mainly contributed by the electron transition from LUMO→HOMO; the electrons in the LUMO orbitals are mainly distributed on 2-(2′-hydroxyphenyl)-benzothiazole, and the electrons in the HOMO orbitals are concentrated on the dimethylphosphinothionyl group. For this reason, the non-radiative emission of PBT can be attributed to the charge separation nature of the S_1_ state. As there is a transfer of excited electrons from the electron donor 2-(2′-hydroxyphenyl)-benzothiazole to the electron acceptor dimethylphosphinothionyl group, the fluorescence quenching of PBT can be attributed to the PET mechanism.

As shown on the right side of Figure 7, the main absorption peak of the product HBT-Enol1 can be attributed to the S_0_-S_1_ transition, which is mainly contributed by the electron transition between HOMO and LUMO. The electron distributions in the HOMO and LUMO orbitals of HBT-Enol1 are mainly localized on the whole HBT-Enol1 and have the characteristics of π-orbitals, indicating that the S_0_-S_1_ transition is a local excitation process with the characteristics of π-transition. Therefore, HBT-Enol1 has strong absorption properties. HBT-Enol1 excitation undergoes a proton transfer process to the HBT-Keto conformation, then HBT-Keto undergoes a radiative transition back to S_0_, which is accompanied by fluorescence. It is worth noting that the HBT-Keto undergoes a partial transfer of electrons from the LUMO to the HOMO orbitals, thus the oscillator strength is relatively small.

## 3. Computational Details

The system was systematically investigated using the DFT/TDDFT method in the Gaussian 16 software package [53]. The B3LYP-D3BJ functional (Becke’s three-parameter hybrid exchange function related to the Lee-Yang-Parr gradient correction [54], complemented by Grimme’s D3 dispersion correction and the Beck-Johnson damping function [55]) and the def-TZVP basis set are used to study the system properties in the ground and excited states [56,57]. In order to better describe this system, we chose the IEF-PCM solvation model to simulate the solvent environment in which the system is exposed and chose water as the solvent [58]. No restriction on molecular symmetry, bond lengths, bond angles or dihedral angles was imposed during the conformational optimization process. All the optimized configurations of the ground and excited states are corroborated by frequency analysis to be local minima on the potential energy surfaces without imaginary frequencies. All optimized transition state configurations were found to have one and only one imaginary frequency by frequency analysis, and the correctness of the transition state structure was further confirmed by intrinsic reaction coordinate (IRC) calculations. All Gibbs free energies were calculated at 298.15 K temperature. On the basis of the optimized structure, the hydrogen bond parameters, including bond length and bond angle, were determined. Absorption, fluorescence and infrared spectroscopic analyses were carried out based on the optimized structures in the S_0_ and S_1_ states. Potential energy profiles were obtained from bond length scans with a step size of 0.1 Å. The Multiwfn program was used for bond order analysis [59].

## 4. Conclusions

In summary, the fluorescence sensing mechanism of probes PBT and F^−^ was investigated in this paper using the DFT/TDDFT method. The fluorescence emission spectra of the HBT-Keto are consistent with the experimental result, therefore the fluorescence observed experimentally is attributed to the HBT-Keto configuration. The low reaction potential barrier (13.94 kcal/mol) between F^−^ and PBT indicates that PBT has a good reaction rate for F^−^. Bond lengths, bond angles, infrared vibrational spectra and changes in hydrogen bond charges for the S_1_ state indicate that the enhanced intramolecular hydrogen bond in the excited state promotes the ESIPT process. The decrease in the energy barrier further evidences the occurrence of ESIPT. FMO analysis confirms that PBT undergoes significant charge transfer, so PBT is non-fluorescent. While, HBT-Keto undergoes an ESIPT process resulting in strong fluorescence emission. From the above analysis, the addition of F^−^ causes the probe to undergo a cleavage reaction, which in turn exposes the hydrogen bond and opens up the possibility of an ESIPT process occurring. This work not only reasonably explains the phenomenon observed in the experiment, but also elaborates the sensing mechanism for the detection of F^−^ based on the PET and ESIPT mechanisms, providing a theoretical basis and support for further experiments.

## Figures and Tables

**Table 1:** Important bond length (Å) and bond angle (°) data related to the hydrogen bond of HBT-Enol1, HBT-Keto in the ground state and excited state.

	HBT-Enol1		HBT-Keto	
	S_0_	S_1_	S_0_	S_1_
O-H_1_	0.994	1.017	1.633	1.871
H_1_⋯N	1.718	1.638	1.053	1.024
δ(O-H_1_⋯N)	147.2	150.2	139.4	130.9

**Table 2:** Theoretical and experimental electronic spectral data for PBT, HBT-Enol1 and HBT-Keto.

	Electronic Transition	Wave Length (nm)	Energy (eV)	f ^a^	Contrib ^b^	CI ^c^	Exp ^d^ (nm)
PBT							
Absorption	S_0_→S_2_	299.79	4.1357	0.0845	H-1→L	0.63	~303
Emission	S_0_→S_1_	637.18	1.9458	0.0203	L→H	0.99	
HBT-Enol1							
Absorption	S_0_→S_1_	332.97	3.7236	0.5117	H→L	0.96	~335
HBT-Keto							
Emission	S_1_→S_0_	495.85	2.5004	0.4520	L→H	0.99	~470

^a^ Oscillator strength. ^b^ H, highest occupied molecular orbital (HOMO); L, lowest unoccupied molecular orbital (LUMO). ^c^ The CI coefficients are in absolute values. ^d^ The experimental absorption spectra data from [30].

## Data Availability

Data are contained within the article and Supplementary Materials.

## Supplementary Materials

The following supporting information can be downloaded at: https://www.mdpi.com/article/10.3390/molecules29071541/s1.
