# Supplementary material for: TDDFT Study on the ESIPT Properties of 2-(2′-Hydroxyphenyl)-Benzothiazole and Sensing Mechanism of a Derived Fluorescent Probe for Fluoride Ion
Source: Molecules. 2024 Mar 29;29(7):1541. doi: 10.3390/molecules29071541 (PMC11013366; doi:10.3390/molecules29071541)
Supplement: Supplementary file 1 [file molecules-29-01541-s001.zip › molecules-2931236-supplementary.pdf]

Supporting Information (SI) for:

TDDFT study on the ESIPT properties of 2-(2'-hydroxyphenyl)-benzothiazole and sensing mechanism of a derived fluorescent probe for fluoride ion

Tingting Wang [1], Meiheng Lv [1,2,*], Yuhang Zhang [1], Yue Gao [1], Zexu Cai [1], Yifan Zhang [1], Jiaqi Song [1], Jianyong Liu [2,*], Hang Yin [2] and Fangjian Shang [3]

1 College of Science, Shenyang University of Chemical Technology, Shenyang 110142, China
2 Research Center of Advanced Biological Manufacture, Dalian National Laboratory for Clean Energy, Dalian Institute of Chemical Physics, Chinese Academy of Sciences, Dalian 116023, China
3 College of Aeronautical Engineering, Binzhou University, Binzhou 256603, China
* Correspondence: mhlv@syuct.edu.cn (M.L.); beam@dicp.ac.cn (J.L.)

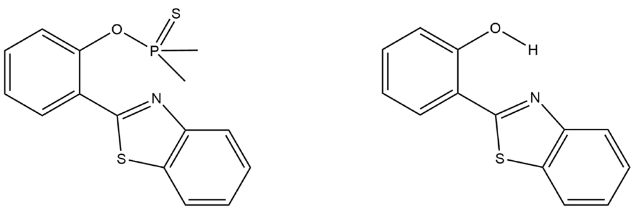

PBT HBT

Figure. S1 The chemical structures of PBT and HBT.

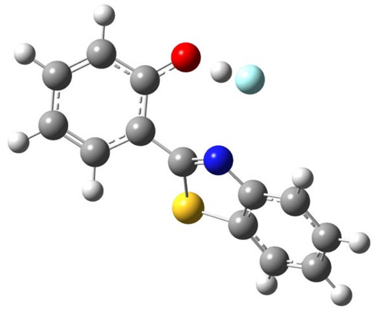

Figure. S2 The optimized ground state geometrical configuration of HBT-F.

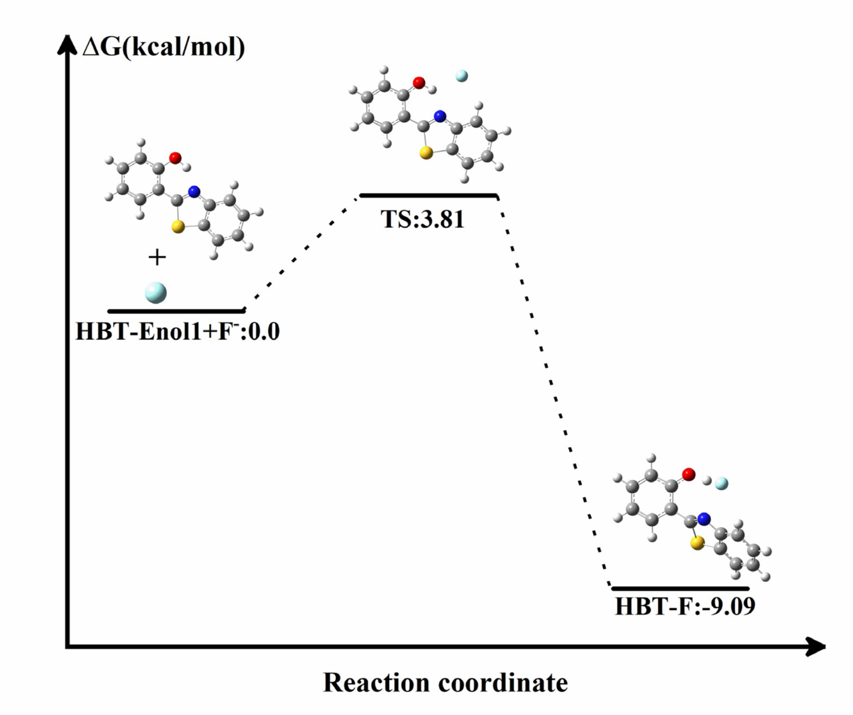

Figure. S3 Gibbs free energy change (kcal/mol) for the HBT-Enol1+F^- recognition process.

IM: intermediate, TS: transition state

Table S1 The natural population analysis of O and N atoms for HBT-Enol1 at the S_0 and S_1 states.

	S_0	S_1
O	-0.662	-0.656
N	-0.486	-0.496

Coordinates of PBT

C	-3.70138300	2.28023700	0.46570200
C	-2.70275000	3.20018300	0.16137700
C	-1.37921600	2.78943400	0.11319000
C	-1.01681300	1.46017400	0.36964200
C	-2.04155700	0.54641000	0.66674700
C	-3.36779300	0.95514700	0.71603000
C	0.39055600	1.05767600	0.30077500
S	1.45405900	1.82031700	-0.92008700
C	2.76529700	0.81426600	-0.33722800
C	2.29636900	-0.00069600	0.71680600
N	0.96388000	0.16923700	1.04445200
C	4.08502800	0.74837600	-0.77448900
C	4.93617500	-0.15147400	-0.14715200
C	4.48309900	-0.96690400	0.89943400
C	3.17039200	-0.89804300	1.33725700
H	-4.73715700	2.59030500	0.50906800
H	-2.95232400	4.23484900	-0.03187100
H	-0.60339800	3.51126000	-0.10645300
H	-4.12862300	0.22245200	0.94948100
H	4.43931600	1.37573700	-1.58118900
H	5.96586800	-0.22265200	-0.47269200
H	5.16917500	-1.65889000	1.37020000
H	2.81248300	-1.52014600	2.14711000
O	-1.75791500	-0.77148500	0.95463800
P	-1.52741200	-1.95488700	-0.20166800
S	-0.91295800	-1.29851000	-1.95870000
C	-0.37304800	-3.03395400	0.68826200
H	-0.77516400	-3.26887100	1.67347100
H	-0.23562300	-3.94935900	0.11475400
H	0.57415100	-2.50979300	0.78833100
C	-3.14138100	-2.79503100	-0.24818200
H	-3.87353300	-2.12561000	-0.69700800
H	-3.05452900	-3.69323800	-0.85839900
H	-3.44876000	-3.05922200	0.76351200

Coordinates of HA^-

C	-4.51807000	-0.26669000	0.00011900
C	-3.72867000	-1.43261700	0.00038500
C	-2.35861900	-1.29592900	0.00035800
C	-1.70132000	-0.04550300	0.00006200
C	-2.50023200	1.18165700	-0.00026400
C	-3.93113200	0.97289200	-0.00017800
C	-0.25706200	-0.02232500	0.00006000
S	0.65413700	-1.61877200	-0.00038800
C	2.15835600	-0.71819200	-0.00015000
C	1.87550000	0.66608800	0.00022000
N	0.53884000	1.00536400	0.00032000
C	3.46433200	-1.19426000	-0.00027500
C	4.50518100	-0.27040300	-0.00003400
C	4.24080500	1.10388400	0.00033100
C	2.93589700	1.57772700	0.00046000
H	-5.59998100	-0.34968500	0.00015400
H	-4.18485000	-2.41371300	0.00063800
H	-1.75961800	-2.19970300	0.00063000
H	-4.54026900	1.87017700	-0.00037700
H	3.67081600	-2.25654500	-0.00055500
H	5.52911500	-0.62152100	-0.00013100
H	5.06469400	1.80628000	0.00051500
H	2.72449400	2.63939400	0.00074600
O	-2.03153500	2.35626800	-0.00052600

Coordinates of HA-F

C	4.47386200	-0.47583900	-0.37537900
C	3.71534700	-1.65006000	-0.41196200
C	2.33719500	-1.55179800	-0.31426200
C	1.69015500	-0.31423900	-0.18539900
C	2.45623500	0.89184600	-0.09393000
C	3.86273900	0.75444000	-0.22136000
C	0.22986500	-0.27791300	-0.16633000
S	-0.66424100	-1.43661800	0.88246300
C	-2.16548400	-0.74644700	0.29451400
C	-1.87210200	0.29147500	-0.61893500
N	-0.52745500	0.51391700	-0.85227400
C	-3.47716900	-1.08633700	0.61190200
C	-4.50476100	-0.37060500	0.01038800
C	-4.22815300	0.66212300	-0.89538600
C	-2.92124100	0.99710200	-1.21494400
H	5.55341100	-0.52761100	-0.46060500
H	4.19046300	-2.61522400	-0.52737100
H	1.73211800	-2.44927500	-0.37133900
H	4.45429800	1.66085700	-0.17015900
H	-3.69432100	-1.88316700	1.31074900
H	-5.53230500	-0.61543800	0.24665600
H	-5.04657200	1.20494200	-1.35060600
H	-2.69910800	1.79238400	-1.91463100
O	1.93791200	2.07118700	0.11265100
H	0.91017000	2.18828000	0.91743600
F	0.15218600	2.36896800	1.63907500

Coordinates of HBT-Enol1

C	4.50546800	-0.09715000	-0.00006400
C	3.84139800	-1.32692500	-0.00019900
C	2.46012700	-1.35044400	-0.00020900
C	1.70710400	-0.16532400	-0.00010600
C	2.39399500	1.07486000	0.00005500
C	3.79120200	1.08825900	0.00008500
C	0.25584400	-0.18610200	-0.00021500
S	-0.68092900	-1.70143300	0.00022200
C	-2.15501000	-0.74328400	0.00006600
C	-1.82981400	0.62886900	-0.00015800
N	-0.47441500	0.89588600	-0.00022700
C	-3.47647200	-1.17635300	0.00018700
C	-4.47757600	-0.21355000	0.00005000
C	-4.16622300	1.15278600	-0.00017100
C	-2.84911300	1.58382200	-0.00024100
H	5.58759600	-0.06656300	0.00002000
H	4.40078400	-2.25240000	-0.00033000
H	1.94563700	-2.30334100	-0.00029400
H	4.29509300	2.04590600	0.00013500
H	-3.72054300	-2.23001400	0.00037800
H	-5.51358500	-0.52612900	0.00011800
H	-4.96665900	1.88098800	-0.00025000
H	-2.59905800	2.63646500	-0.00040200
O	1.75090200	2.25928300	0.00046100
H	0.77371100	2.07576100	0.00050000

Coordinates of HBT-Enol2

C	-4.50065300	-0.27474200	0.00021800
C	-3.73663600	-1.43962000	0.00063300
C	-2.35775100	-1.34231300	0.00058600
C	-1.68747000	-0.10650900	0.00012500
C	-2.48125000	1.06310900	-0.00031700
C	-3.87474900	0.95993400	-0.00024400
C	-0.22485900	-0.07959300	0.00010200
S	0.68216300	-1.64340900	-0.00064700
C	2.17497200	-0.72776300	-0.00028100
C	1.87863800	0.65214600	0.00041800
N	0.53398300	0.96857300	0.00061100
C	3.48693500	-1.19104800	-0.00055800
C	4.51076900	-0.25244700	-0.00013500
C	4.23127200	1.12100000	0.00056100
C	2.92382300	1.58063300	0.00083900
H	-5.58167300	-0.32635200	0.00026300
H	-4.21144900	-2.41116800	0.00100900
H	-1.77693000	-2.25514800	0.00097900
H	-4.46523800	1.86903300	-0.00058000
H	3.70666400	-2.25025300	-0.00108400
H	5.53934800	-0.58922100	-0.00034100
H	5.04842200	1.83061500	0.00088300
H	2.69778600	2.63887400	0.00137600
O	-1.88717000	2.28311500	-0.00086200
H	-2.57031900	2.96652600	-0.00121500

Coordinates of HBT-Keto

C	4.50496600	-0.01415300	0.00054600
C	3.88253800	-1.28358300	-0.00006900
C	2.51406900	-1.35408600	-0.00056100
C	1.71846900	-0.18408900	-0.00054100
C	2.33968700	1.13166200	0.00007700
C	3.77095700	1.14398300	0.00071700
C	0.30613700	-0.25571800	-0.00058600
S	-0.67076800	-1.71870400	0.00003900
C	-2.14472100	-0.73910900	0.00026800
C	-1.82647400	0.62676700	-0.00010600
N	-0.45958800	0.83716200	-0.00069400
C	-3.46509600	-1.16794600	0.00053700
C	-4.46424300	-0.20125400	0.00045100
C	-4.14816100	1.16157400	0.00002700
C	-2.82879600	1.59230200	-0.00032800
H	5.58732100	0.04303300	0.00100200
H	4.48053000	-2.18451500	-0.00031200
H	2.02740700	-2.32284600	-0.00103800
H	4.25652800	2.11198900	0.00115600
H	-3.71042600	-2.22100700	0.00076500
H	-5.50078800	-0.51072800	0.00072100
H	-4.94444000	1.89384300	-0.00007000
H	-2.57837600	2.64433000	-0.00074600
O	1.65939700	2.21472200	0.00007700
H	0.10048200	1.72914800	-0.00044600
